# Development and evaluation of a social marketing campaign to address methamphetamine use in Los Angeles County

**DOI:** 10.1186/s12889-022-14180-y

**Published:** 2022-09-22

**Authors:** Deborah Neffa-Creech, Aaron Plant, Jorge A. Montoya, Rangell Oruga, Elizabeth A. Kilgore, Renee Fraser, Lello Tesema

**Affiliations:** 1 Sentient Research, 231 North Walnuthaven Drive, West Covina, CA 91790 USA; 2 Division of Substance Abuse Prevention and Control, Los Angeles County Department of Public Health, 1000 South Fremont Avenue, Bldg A-9 East, 3rd Floor, Alhambra, CA 91803 USA; 3 Fraser Communications, 1631 Pontius Avenue, Los Angeles, CA 90025 USA

**Keywords:** Social marketing, Mass media campaign, Campaign development, Evaluation, Methamphetamine, Prevention, Treatment

## Abstract

**Background:**

This study describes the development and impact of a social marketing campaign in early 2020 intended to prevent and reduce methamphetamine use in Los Angeles County (LAC). We used social marketing principles and the transtheoretical model to design the campaign, which was intended to avoid stigmatization of methamphetamine users and communicate compassion, empathy, and support.

**Methods:**

To evaluate its impact, we collected cross-sectional online survey data post-campaign (*n* = 1,873) from LAC residents in population segments considered higher risk for methamphetamine use. We examine associations between campaign exposure and outcomes using bivariate analyses and binary logistic regression models, which control for the impact of the COVID-19 pandemic on methamphetamine use or likelihood of use.

**Results:**

The analyses revealed that campaign exposure was associated with having more negative attitudes toward methamphetamine, calling LAC’s substance abuse service helpline, using methamphetamine fewer days, and considering abstaining. Frequency of exposure to campaign advertisements was positively associated with calling the helpline, suggesting a campaign dose effect. COVID-19-related factors were associated with using methamphetamine in the past 30 days.

**Conclusions:**

Social marketing campaigns hold promise for impacting methamphetamine prevention and cessation behaviors. This study adds to the limited literature on mass marketing interventions to address this major health issue.

**Supplementary Information:**

The online version contains supplementary material available at 10.1186/s12889-022-14180-y.

## Introduction

Methamphetamine is a highly addictive and widely available illicit stimulant associated with a range of negative physical and mental health outcomes [1–5]. An estimated 15,939,000 U.S. adults (6.4%) have used methamphetamine, including 1,958,000 (0.8%) in the past year [6]. Over one million U.S. adults are estimated to have a methamphetamine use disorder [6]. Use of methamphetamine also appears to be a growing problem, as evidenced by large increases in treatment admissions over the past decade [7]. Overdose deaths in the U.S. involving psychostimulants such as methamphetamine increased nearly five-fold from 2012–2018 [8].

Methamphetamine use in Los Angeles County (LAC) is a major public health issue with an increasing impact over the past several decades [9]. A 2019 representative survey found that 79,000 (1%) of LAC adults had used methamphetamine in the past year [10]. The LAC Department of Public Health reported that the number of hospitalizations and emergency room visits due to methamphetamine increased by over 600% from 2008–2017 [11]. In 2019, there were 576 deaths due to methamphetamine, a 1,240% increase since 2008 [12]. Methamphetamine overdose deaths represented 45% of all drug overdose deaths in LAC in 2019 [12].

Mass media campaigns have frequently been implemented in an attempt to address misuse and abuse of substances, including tobacco, alcohol, and illicit drugs, among a wide range of populations and settings [13–16]. Some of the strongest outcomes have been observed in anti-tobacco campaigns [13, 14]. However, systematic reviews of campaigns have found mixed results regarding substance use behaviors more broadly [16–18].

Despite the scale of the issue, very few methamphetamine campaigns have been evaluated with results published. A 2005 study of a New York City campaign found improvements in attitudes and intentions around methamphetamine use but did not assess behaviors [19]. The few studies of methamphetamine campaigns reporting behavior outcomes have had mixed results. Pooled estimates of effects from five studies of the Meth Project, a large-scale and widely implemented methamphetamine prevention campaign, found a reduction in methamphetamine use in the past year among youth ages 12–17, but not among older age groups [20]. Another Meth Project study found evidence of a reduction in methamphetamine use only among White high school students [21], while a third evaluation study found no impact on behavior [22].

The Los Angeles County Department of Public Health (LACDPH) has had success with social marketing campaigns [23–25], including those targeting substance use. For example, an evaluation of its 2013 campaign to reduce tobacco use among sexual minority persons found an association between campaign exposure and quitting intentions and taking steps to quit [26]. In 2020, LACDPH launched a multimedia campaign to address methamphetamine use among several population segments in LAC considered to be at higher risk. This paper details program planning efforts and outcomes from the campaign evaluation.

## Methods

LACDPH funded a multimedia campaign to prevent use of methamphetamine and to encourage those who use the drug to reduce use, practice harm reduction, or abstain. To maximize the campaign’s impact and value, we focused on population segments at higher risk for methamphetamine use based on County surveillance data and secondary research with community stakeholders working in methamphetamine prevention and treatment. These segments included: individuals living in LAC zip codes with the highest number of overdoses due to methamphetamine; men who have sex with men, or MSM [5]; individuals recently experiencing homelessness [7] or prolonged unemployment [7]; and other groups, such as those in specific job categories known to be at elevated risk (see Table 1 for details of those in higher-risk segments). The age range for most segments was 18–35, which is consistent with County-wide age-related data for methamphetamine use [11]. For MSM, we targeted ages 18 and older as community stakeholders indicated methamphetamine use among a wider age range for this segment.

**Table 1:** Differences in participant background characteristics and campaign outcomes by exposure to *Meth-Free L.A. County* campaign

	No	Overall	Exposed	Not exposed	Bivariate estimate of exposure
		% or *M* (SD)			OR (95% CI) or t-test
Race/ethnicity					
White (non-Hispanic)	650	35.4	27.7	72.3	1 (Ref)
Black (non-Hispanic)	235	12.8	27.7	72.3	1.00 (0.71, 1.39)
Hispanic or Latinx	539	29.4	23.0	77.0	0.78 (0.60, 1.02)
Asian (non-Hispanic)	179	9.8	13.4	86.6	0.40*** (0.25, 0.64)
Mixed race^a^	205	11.2	24.4	75.6	0.84 (0.59, 1.21)
Other (non-Hispanic)	26	1.4	19.2	80.8	0.62 (0.23, 1.67)
Gender					
Female	755	40.3	20.4	79.6	1 (Ref)
Male	1065	56.9	26.9	73.1	1.44*** (1.15, 1.80)
Other or unsure	53	2.8	32.1	67.9	1.84* (1.01, 3.37)
Age (range: 18–70)	1873	30.7 (9.86)	30.6 (9.50)	30.7 (9.98)	*t*(1871) = 0.28
Education					
High school degree or less	354	18.9	26.0	74.0	1 (Ref)
Some college or trade school	508	27.1	22.6	77.4	0.83 (0.61, 1.14)
College degree	691	36.9	24.0	76.0	0.90 (0.67, 1.21)
Graduate work or degree	320	17.1	26.6	73.4	1.03 (0.73, 1.45)
Annual household income					
≤ $30,000	634	35.8	25.4	74.6	1 (Ref)
$30,001-$60,000	483	27.3	23.0	77.0	0.88 (0.66, 1.16)
$60,001 or higher	653	36.9	25.9	74.1	1.03 (0.80, 1.32)
Meth use history					
Never	1485	81.1	20.8	79.2	1 (Ref)
Over 12 months ago	176	9.6	34.1	65.9	1.97*** (1.41, 2.75)
Over 30 days-12 months ago	63	3.4	49.2	50.8	3.69*** (2.21, 6.14)
In the past 30 days	107	5.8	43.0	57.0	2.87*** (1.92, 4.29)
Men who have sex with men, ages 18–70					
No	1202	64.2	22.9	77.1	1 (Ref)
Yes	671	35.8	27.3	72.7	1.26* (1.02, 1.57)
Adults in higher-risk jobs^b^, ages 18–35					
No	926	49.4	21.2	78.8	1 (Ref)
Yes	947	50.6	27.7	72.3	1.42*** (1.15, 1.76)
Adults who live in higher-risk zip codes, ages 18–35					
No	1019	54.4	23.7	76.3	1 (Ref)
Yes	854	45.6	25.4	74.6	1.10 (0.89, 1.36)
Adults unemployed at least 9 months in the past year, ages 18–35					
No	1472	78.6	24.8	75.2	1 (Ref)
Yes	401	21.4	23.2	76.8	0.92 (0.71, 1.19)
Adults who experienced homelessness in the past year, ages 18–35					
No	1673	89.3	22.4	77.6	1 (Ref)
Yes	200	10.7	41.5	58.5	2.45*** (1.81, 3.33)
COVID-19-related factors scale (5 = *greater likelihood of using or actual use of meth)*	1518	3.57 (0.78)	3.62 (0.78)	3.55 (0.78)	*t*(1516) = -1.39
Negative attitudes towards meth use (5 = *more negative attitudes*)	1873	3.84 (0.61)	3.90 (0.65)	3.83 (0.60)	*t*(1871)* = -2.20
Concerned about the impact of meth in the community (5 = *very concerned*)	1873	3.25 (1.31)	3.41 (1.33)	3.19 (1.30)	*t*(1871)** = -3.14
Has discussed meth use with others					
No	800	42.7	16.7	83.3	1 (Ref)
Yes	1073	57.3	30.2	69.8	2.15*** (1.71, 2.70)
Has looked online for meth prevention or treatment information					
No	1257	67.1	18.6	81.4	1 (Ref)
Yes	616	32.9	36.4	63.6	2.50*** (2.01, 3.10)
Has heard of SASH					
No	1270	67.8	19.5	80.5	1 (Ref)
Yes	603	32.2	34.8	65.2	2.20*** (1.77, 2.73)
Has ever called SASH					
No	1746	93.2	21.3	78.7	1 (Ref)
Yes	127	6.8	67.7	32.3	7.75*** (5.25, 11.43)
Used meth the past 30 days					
No	1724	94.3	43.0	57.0	1 (Ref)
Yes	107	5.7	23.2	76.8	2.50*** (1.67, 3.72)
Number of days used meth in the past 30 days					
1–9 days	35	32.7	65.7	34.3	1 (Ref)
10 days-everyday	72	67.3	31.9	68.1	0.24*** (0.10, 0.58)
Is currently considering quitting meth use					
No	40	38.5	35.0	65.0	1 (Ref)
Yes	64	61.5	48.4	51.6	1.74 (0.77, 3.94)
When plans to quit meth use					
Over 30 days or unsure	38	59.4	34.2	65.8	1 (Ref)
In the next 30 days	26	40.6	69.2	30.8	4.33** (1.48, 12.60)

*N* 1,873, *OR* Odds Ratio, *CI* Confidence Interval. Those who declined responses are excluded

^a^Mixed race is a mutually exclusive category

^b^Respondents in higher-risk jobs work: more than 12 h a day; in two or more jobs; 2nd and/or 3rd shifts; in the entertainment industry or are Uber/Lyft/cab drivers; bar, restaurant, customer service, or other hospitality workers; truck drivers, movers; janitorial workers; construction workers; house painters; factory/manufacturing/warehouse workers; and/or agriculture workers

^*^p ≤ .05

^**^p ≤ .01

^***^p ≤ .001

### Social marketing principles and formative research

We applied the principles of social marketing to develop the campaign. This included a focus on behavior change outcomes; expert and audience research to develop the campaign messaging and brand and to determine promotion channels; audience segmentation; and application of behavior change theory [27]. The creative development process began with a literature review of methamphetamine use, along with a comparative analysis of prior methamphetamine campaigns. Next, we conducted 15 in-depth interviews with diverse local experts, including clinicians, outreach staff working in methamphetamine treatment, and university researchers in the field. These interviews explored the context of methamphetamine use in LAC, including population segments most at risk, and facilitators and barriers to recovery. A major theme of the interviews was the need to develop messages that were empathic and would not further marginalize and stigmatize people who use methamphetamine, as participants felt this would be counterproductive.

Formative research continued with three, 1.5-h focus groups (two with females and one with males; total *n* = 23) to inform prevention messaging. LAC residents who had never used methamphetamine, but who knew at least one person who currently uses the drug, were asked about methamphetamine knowledge, beliefs, and attitudes; consequences of use; reasons why they might or might not try methamphetamine; feedback on past methamphetamine campaigns; and promotion channels for the new campaign. We then conducted one-hour interviews (*n* = 15) in person or by telephone with male and female LAC residents who regularly use or recently quit using methamphetamine to inform harm reduction and cessation messaging. These interviews covered participant experiences with and attitudes toward methamphetamine; barriers and facilitators to using less or abstaining; feedback on past campaigns; and promotion channels. Results from the interviews and focus groups were used to create several campaign brand and messaging concepts, which were tested in a second round of two, 1.5-h focus groups (*n* = 15), one with males and the other with females.

### Development and dissemination of campaign advertisements

**Fig. 1:**
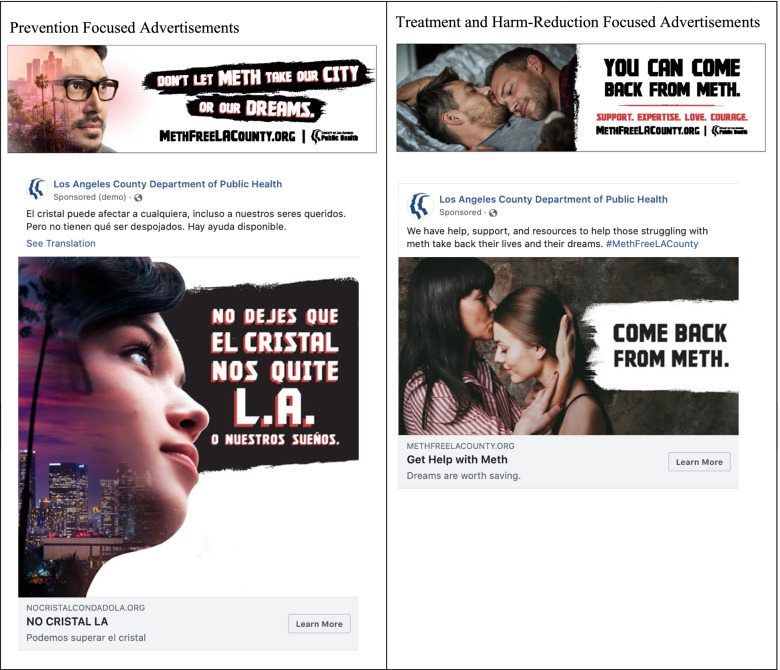
*Meth-free L.A. County* campaign print and social media advertisement examples, in English and Spanish

Concept testing findings were used to develop the final campaign brand, *Meth-Free L.A. County*. The first set of advertisements focused on prevention messaging among residents at higher risk of using methamphetamine but who have never tried the drug or have tried but do not currently use it. These advertisements highlighted the potential negative impacts of methamphetamine use on personal aspirations, as well on the general quality of life in Los Angeles (see Fig. 1). The second set of advertisements promoted harm reduction and treatment to current methamphetamine users. These advertisements encouraged reduction or abstinence by emphasizing the positive impact that cessation could have on their relationships and overall well-being (Fig. 1). The entire campaign was designed to be compassionate, empathetic, and supportive of individuals and their health. This approach was intended to avoid further cultural stigmatization of methamphetamine users, as our formative research interviews and prior studies indicated that negative depictions could hinder treatment-seeking behaviors or abstinence [28–30].

The campaign’s messages incorporated constructs from the transtheoretical model, which posits that health behavior change is a progression through stages (e.g., precontemplation, contemplation, preparation, action, maintenance, and termination; [31]). *Meth-Free L.A. County* campaign advertisements were designed to reach residents at different stages of change and to encourage movement across stages. For example, residents in the contemplation stage may be aware that a problem exists and are considering reducing or abstaining from methamphetamine use but have not yet taken action. Similarly, residents in the action stage may modify their behavior by calling the LAC Substance Abuse Service Helpline (SASH) and seeking substance use treatment.

Print, audio, and video *Meth-Free L.A. County* advertisements in English and Spanish ran in LAC for 10 weeks between February and April 2020 across multiple channels (e.g., high-profile outdoor banners, radio and streaming audio, and social media platforms).

### Data Collection

We conducted an online survey of residents five days after the campaign ended to assess the impact of campaign exposure on a range of methamphetamine use-related attitudes and behaviors. We used a purposive sampling approach that targeted adults who currently use methamphetamine or are at higher risk of doing so (i.e., those in the population segments of interest; see Table 1). Participants were recruited from May 5–20, 2020, using Dynata, an online research firm that maintains a panel of several million U.S. adults, and paid advertisements on social media and geospatial networking applications.

### Measures

#### Demographics and background characteristics

A range of participant characteristics were assessed, including those to estimate potential risk for methamphetamine use, such as current housing status or employment (see Table 1).

#### Campaign exposure

Both unaided and aided campaign awareness were assessed. Respondents were first asked, via an open-ended question, if they could recall advertisements, publicity, events, or marketing that provided information about methamphetamine during the last six months. Those who mentioned the *Meth-Free L.A. County* campaign or its advertisements by name were considered to have unaided awareness. Next, respondents were presented with several radio, television, and print advertisements. Participants who reported seeing or hearing any of these advertisements in the past three months were considered to have aided awareness. Aided and unaided awareness were combined into a single exposure variable for analysis (0 = no campaign exposure; 1 = campaign exposure).

#### Campaign exposure level

Level of campaign exposure was assessed by asking respondents to estimate the number of times they saw or heard a *Meth-Free L.A. County* campaign advertisement in the past three months. We used a median split (one to four times versus five or more times) to identify those with lower-level (*n* = 239) and higher-level (*n* = 209) exposure and to create a tiered variable: no campaign exposure, lower-level exposure, and higher-level exposure.

#### Perceived impact of campaign advertisements

All participants were shown campaign advertisements in the survey (to capture aided awareness). After viewing them, they were asked a series of agreement statements about perceived impact of the advertisements on personal methamphetamine-related awareness, concern, and intentions (see Additional file 1). As such, participants who both self-reported campaign exposure and no exposure reviewed campaign advertisements in the survey and self-reported on their perceived impact. Mean values were calculated for all statements by self-reported campaign exposure, methamphetamine use history, and the other population segments of interest.

#### Attitudes and concern related to methamphetamine use

Attitudes were assessed using a series of 14 agreement statements on a five-point scale (1 = strongly disagree, 5 = strongly agree). Five of these statements loaded onto one component in a factor analysis using a principal component analysis extraction method and Varimax rotation. The five statements were averaged into a scale called *negative attitudes towards methamphetamine use* (α = 0.63): “methamphetamine is a highly addictive drug,” “methamphetamine is one of the most dangerous drugs,” “anyone could become addicted to methamphetamine,” “methamphetamine addiction is an illness or disease,” and “methamphetamine use is one of the major issues in my community.” We also asked respondents to rate their level of concern about methamphetamine in their community using a five-point scale (1 = not at all concerned, 5 = very concerned).

#### Information seeking and discussing methamphetamine with others

We asked respondents whether they have ever talked about methamphetamine with anyone or looked online for methamphetamine use prevention or treatment information (0 = no, 1 = yes).

#### Helpline (SASH) awareness and use

Respondents were asked whether they had heard of the SASH helpline or had ever called SASH (0 = no, 1 = yes).

Methamphetamine use, intentions to quit, and steps to quit*.* We asked respondents whether they have ever used methamphetamine. Those who said “yes” were asked how recently they had used it (in the past 30 days, between 30 days and 12 months ago, or more than 12 months ago). Those who used methamphetamine in the past 12 months were asked if they had ever taken steps to quit, and those who used methamphetamine in the past 30 days were also asked: the number of days in the past month they had used, if they currently have intentions to quit, and whether they intend to quit in the next 30 days.

#### COVID-19 factors related to methamphetamine use or likelihood to use

The last six weeks of the campaign overlapped with the COVID-19 pandemic [32] and with LAC's first “safer at home” ordinance [33]. As social isolation and stress might affect methamphetamine use behaviors, we asked, using a five-point scale (1 = strongly decreased, 5 = strongly increased), whether the following had impacted respondents’ likelihood to use or actual use of methamphetamine: anxiety about becoming infected with COVID-19; anxiety that someone they care about will be infected with COVID-19; stress caused by the “safer at home” ordinance; feelings of isolation; feelings of boredom or having too much free time; and general anxiety about the COVID-19 pandemic. All items loaded strongly in a principal component analysis and were averaged into a scale (α = 0.88). “Not applicable” responses (19%) were excluded from the scale.

### Analyses

We used descriptive statistics, bivariate analyses (e.g., logistic regression and t-tests) and five multivariable binary logistic regression models. The models assessed the effect of campaign exposure on primary outcomes while controlling for participant background characteristics, methamphetamine use behaviors, and the COVID-19-related factors scale. Outcome measures were dichotomized as 0 and 1, and we used a median split to examine composite attitude scores. We used SPSS (version 27) to perform analyses.

We employed several strategies to maximize the quality of the online survey data, including discarding responses from duplicate IP addresses and those with similar email addresses. Further, we omitted responses completed more quickly than deemed reasonable, which we determined by having three individuals take the survey as quickly as possible while reading all questions.

## Results

We retained 80% of responses (*n* = 1,873) in the analytic sample. Most participants were male (56.9%), and the average age was 30.7 (Table 1). The most common race/ethnicity categories reported were non-Hispanic White (35.4%) and Hispanic or Latinx (29.4%). Nineteen percent of participants reported ever using methamphetamine, and 5.8% reported using it within the past 30 days.

A total of 24.5% of respondents self-reported being exposed to the *Meth-Free L.A. County* campaign in the past three months (3% had unaided awareness and 24.3% had aided awareness). Those exposed reported seeing or hearing campaign advertisements on average eight times in the past three months. Respondents at greater risk of methamphetamine use were more likely to report exposure to the campaign – those who have ever used methamphetamine, in particular in the past 30 days (OR = 2.87; 95% CI = 1.92, 4.29; *p* ≤ 0.001), have experienced homelessness in the past year (OR = 2.45; 95% CI = 1.81, 3.33; *p* ≤ 0.001), work in higher-risk jobs (OR = 1.42; 95% CI = 1.15, 1.76; *p* ≤ 0.001), and identify as MSM (OR = 1.26; 95% CI = 1.02, 1.57; *p* ≤ 0.05).

Participants who either self-reported campaign exposure or no exposure felt that campaign advertisements were impactful (Additional file 1). Overall. they were likely to agree that the advertisements taught them something about methamphetamine (*M* = 3.83, *SD* ± 1.06); increased their concerns about the risks of methamphetamine use (*M* = 3.90, *SD* ± 1.02) and the impact of methamphetamine in the community (*M* = 4.03, *SD* ± 0.96); and informed them of treatment options and resources (*M* = 4.01, *SD* ± 0.93), such as SASH (*M* = 4.02, *SD* ± 0.97).

Respondents with no prior methamphetamine use agreed that the advertisements made them less likely to try it in the future (*M* = 4.17, *SD* ± 1.04). Those who self-reported prior use agreed the advertisements made them think their use of the drug was a problem (*M* = 3.69 *SD* ± 1.20) and made them consider reducing their use (*M* = 3.68, *SD* ± 1.23) or quitting (*M* = 3.82, *SD* ± 1.16). See Additional File 1 for responses among other population segments (e.g., MSM and those in higher-risk zip codes and higher-risk jobs).

The bivariate analyses suggest that campaign exposure was associated with a range of methamphetamine use prevention and reduction outcomes (Table 1). Those exposed scored higher on negative attitudes towards methamphetamine use (*p* ≤ 0.05); reported greater concern about methamphetamine use in the community (*p* ≤ 0.01); and were more likely to discuss methamphetamine with others (*p* ≤ 0.001) and seek methamphetamine-related information online (*p* ≤ 0.001). Those exposed were also more likely to have heard of SASH (*p* ≤ 0.001) and to have ever called SASH (*p* ≤ 0.001). Among those who have used methamphetamine in the past 30 days, exposure was associated with using fewer than 10 days (*p* ≤ 0.001) and with greater intentions to quit using in the next 30 days (*p* ≤ 0.01).

A significant association remained between self-reported campaign exposure and primary outcomes after controlling for demographics and other explanatory variables in the binary logistic regression models (Additional file 2). Specifically, higher-level exposure (reports of seeing or hearing campaign advertisements five or more times in the past three months) was associated with having more negative attitudes towards methamphetamine use (OR = 1.57; 95% CI = 1.09, 2.26; *p* ≤ 0.05), using methamphetamine fewer than 10 days in the past 30 days (OR = 10.43; 95% CI = 3.03, 35.84; *p* ≤ 0.001), and considering quitting methamphetamine use (OR = 14.16; 95% CI = 1.52, 131.83; *p* ≤ 0.05). A campaign dose effect was present for having ever called SASH – those reporting higher-level exposure (OR = 7.57; 95% CI = 4.16, 13.79; *p* ≤ . 001) had greater odds than those reporting lower-level exposure (OR = 5.88; 95% CI = 3.24, 10.66; *p* ≤ 0.001) of having ever called SASH, compared to those reporting no campaign exposure. Campaign exposure was also related to increased odds of methamphetamine use in the past 30 days, indicating the campaign reached its intended audience. The COVID-19-related factors scale was also associated with increased odds of methamphetamine use in the past 30 days.

## Discussion

*Meth-Free L.A. County* was associated with self-reported attitudes and behaviors related to methamphetamine use. These included more negative attitudes toward methamphetamine use, concerns about the impact of methamphetamine in the community, awareness and use of LAC’s substance abuse helpline (SASH), considerations of quitting, and fewer days using in the past 30 days. The campaign achieved a relatively high exposure level (24.5%), given its 10-week duration and dissemination amidst a deluge of public health messages related to the COVID-19 pandemic. The multi-media advertisements successfully reached residents in target segments (range 23.2% to 41.5% reporting exposure; Table 1). Of note is that campaign exposure among those who have used methamphetamine in the past was 39.6% compared to 20.8% among those who have never used it. While a main benefit of mass media campaigns is their ability to reach large numbers of people [17, 18], these programs can be costly. Our evaluation suggests that focusing on population segments at likely higher risk of methamphetamine use was an efficient and effective use of limited campaign funds.

Overall, respondents agreed with statements that *Meth-Free L.A. County* advertisements impacted their awareness, concerns, and intentions related to methamphetamine use and awareness of treatment-related resources. Agreement with statements was consistently higher among those who self-reported campaign exposure (versus no exposure) and prior use of methamphetamine (versus no prior use). These findings may help to explain the associations between campaign exposure and self-reported attitudinal and behavioral outcomes.

The results of our study also suggest there is an association between outcomes and campaign exposure level, or dose. Dose–response effects have been reported in tobacco campaign evaluation studies [14, 29, 34]; however, to our knowledge, this has not been reported for methamphetamine-related campaigns. In our study, a dose–response effect was present for having ever called SASH. Only higher-level campaign exposure was associated with other primary outcomes, an indication of the importance of repeated advertisement exposure and benefits of multi-channel media campaigns. A longer campaign could have led to more exposure and potentially larger impacts.

We used the transtheoretical model’s multiple stages to guide the campaign’s intended outcomes (e.g., concerns about methamphetamine use, information seeking, use of methamphetamine, and taking steps to quit). Self-reported campaign exposure was associated with outcomes at multiple stages of change, further strengthening the results. The findings suggest that *Meth-Free L.A. County* appeared successful at behavioral precursors to methamphetamine use and at behaviors related to cutting down or quitting. This may have been a result of careful campaign planning and a focus on the principles of social marketing, including target audience research and stakeholder engagement. Another factor may have been the campaign’s focus on positive messaging; this contrasts markedly with prior methamphetamine campaigns, which tend to be overtly fear-based [19, 21]. Prior studies on health campaign messaging have demonstrated the promise of hope-based appeals and other messaging strategies meant to evoke positive affect [35].

Our study also controlled for the possible effects of the COVID-19 pandemic, as it coincided with campaign implementation. It is likely that COVID-19-related anxiety, social isolation, and stress potentially exacerbated by the “stay at home” ordinance, impacted usage behaviors. 

### Limitations

This study is not without limitations. Because our study used a post-test-only design, we cannot establish causation between campaign exposure and outcomes. There is no pre-campaign survey for pre-post comparison, and campaign exposure status is based on participant recollection. Measures were self-reported and could have been affected by social desirability bias due to the stigmatized nature of methamphetamine use. However, the online and anonymous nature of the survey likely mitigated this bias. Some analyses might be subject to biases due to the way that measures were captured in the survey. In particular, the outcome measure “ever called SASH” is not time-bound; thus, those who might have called SASH pre-campaign might be more attuned to messaging about methamphetamine. Also, the items in the COVID-19 factors scale ask respondents if COVID-19-related anxiety and stress impacted their use of, or likelihood to use, methamphetamine. This might have introduced a biased positive association between the scale and outcomes related to methamphetamine use in the binary logistic regression models. Lastly, due to budget limitations, the study consisted of English-speaking residents only, meaning we were unable to capture the impact of the Spanish-language campaign advertisements. 

## Conclusions

While social marketing campaigns have been demonstrated effective at improving a range of health behaviors, very few evaluations of methamphetamine campaigns have been published. Results from this evaluation suggests that carefully planned, tailored social marketing campaigns, even those that are relatively brief, may help prevent or reduce methamphetamine use among population segments at higher risk for using. Additionally, our study investigated the impact of dose on campaign outcomes and controlled for potential effects of an unforeseen outside event, the COVID-19 pandemic. Given increases in drug-related morbidity and mortality amidst the pandemic, social marketing campaigns can play a critical role in mitigating individual and community harms related to drug use.

## Supplementary Information

**Additional file 1.** Agreement with perceived impact statements for campaign advertisements by different population segments, *M* (SD).

**Additional file 2.** Associations between campaign exposure level and main outcome variables.

## Data Availability

Fully de-identified data can be made available from the corresponding author on reasonable request (deborah@sentientresearch.net).

## Abbreviations

LAC: Los Angeles County
LACDPH: Los Angeles County Department of Public Health
MSM: Men who have sex with men
SASH: Substance Abuse Service Helpline
