# Supplementary material for: Development and evaluation of a social marketing campaign to address methamphetamine use in Los Angeles County
Source: BMC Public Health. 2022 Sep 22;22:1796. doi: 10.1186/s12889-022-14180-y (PMC9493153; doi:10.1186/s12889-022-14180-y)
Supplement: Supplementary file 1 — Additional file 1. Agreement with perceived impact statements for campaign advertisements by different population segments, M (SD). [file 12889_2022_14180_MOESM1_ESM.docx]

Additional File 1. Agreement with Perceived Impact Statements for Campaign Advertisements by Different Population Segments, *M* (SD)

|  |  |  | **Self-reported campaign exposure** | | **Self-reported past use of meth** | | **Other population segments at greater risk for meth use** | | | | |  |
| --- | --- | --- | --- | --- | --- | --- | --- | --- | --- | --- | --- | --- |
| The campaign ads... | No. | All | Yes | No | Yes | No | MSM | Higher-risk job | Higher-risk zip code | Long unemployment | Homeless in the past year | |
| Taught me something about meth | 1837 | 3.83 (1.06) | 3.93  (1.10) | 3.80  (1.05) | 3.49 (1.20) | 3.92 (1.01) | 3.63 (1.11) | 3.89 (1.05) | 3.93 (1.06) | 3.67 (1.21) | 3.57 (1.23) | |
| Increased my concern about the risks of meth use | 1818 | 3.90 (1.02) | 3.96 (1.05) | 3.89  (1.01) | 3.70 (1.11) | 3.96 (0.99) | 3.78 (1.06) | 3.93 (1.03) | 4.01 (1.00) | 3.74 (1.07) | 3.73 (1.17) | |
| Increased my concern of the impact of meth in the community | 1839 | 4.03 (0.96) | 4.10 (0.93) | 4.00  (0.97) | 3.85 (1.07) | 4.08 (0.93) | 3.91 (1.01) | 4.07 (0.96) | 4.13 (0.94) | 3.95 (1.00) | 3.89 (1.07) | |
| Made me more empathetic toward those who use meth | 1817 | 3.73 (1.01) | 3.90 (1.02) | 3.68  (1.01) | 3.63 (1.16) | 3.76 (0.98) | 3.61 (1.03) | 3.78 (1.03) | 3.86 (1.01) | 3.67 (1.05) | 3.59 (1.20) | |
| Made me aware of SASH | 1817 | 4.02 (0.97) | 4.10 (1.00) | 3.99 (0.96) | 3.88 (1.06) | 4.06 (0.94) | 3.85 (1.01) | 4.05 (0.99) | 4.18 (0.89) | 3.98 (1.00) | 3.87 (1.04) | |
| Made me aware of available treatment options for meth | 1840 | 4.01 (0.93) | 4.13 (0.92) | 3.97  (0.93) | 3.91 (1.02) | 4.04 (0.90) | 3.97 (0.90) | 4.02 (0.96) | 4.07 (0.94) | 3.99 (0.91) | 3.92 (0.90) | |
| Made me less likely to try meth in the future | 1368 | 4.17 (1.04) | 4.16 (1.04) | 4.17 (1.04) | --- | 4.17 (1.04) | 4.01 (1.12) | 4.17 (1.05) | 4.25 (1.01) | 4.26 (1.01) | 3.96 (1.08) | |
| Made me consider quitting (or never starting again) | 289 | 3.82 (1.16) | 4.05 (1.02) | 3.66 (1.22) | 3.82 (1.16) | --- | 3.73 (1.12) | 3.91 (1.21) | 4.09 (1.16) | 3.64 (1.17) | 3.76 (1.26) | |
| Made me think about reducing my meth use | 239 | 3.68 (1.23) | 3.88 (1.18) | 3.53 (1.25) | 3.68 (1.23) | --- | 3.53 (1.21) | 3.79 (1.30) | 3.94 (1.31) | 3.54 (1.22) | 3.77 (1.26) | |
| Made me think my meth use is (or was) a problem | 284 | 3.69 (1.20) | 3.84 (1.17) | 3.59 (1.22) | 3.69 (1.20) | --- | 3.54 (1.21) | 3.80 (1.20) | 4.00 (1.19) | 3.50 (1.19) | 3.71 (1.24) | |

*Notes*. Range is 1 (strongly disagree) to 5 (strongly agree). All participants, regardless of self-reported exposure to the campaign, were shown the campaign advertisements in the survey and asked these statements.
