# Supplementary material for: Development and evaluation of a social marketing campaign to address methamphetamine use in Los Angeles County
Source: BMC Public Health. 2022 Sep 22;22:1796. doi: 10.1186/s12889-022-14180-y (PMC9493153; doi:10.1186/s12889-022-14180-y)
Supplement: Supplementary file 2 — Additional file 2. Associations between campaign exposure level and main outcome variables. [file 12889_2022_14180_MOESM2_ESM.docx]

Additional File 2. Associations Between Campaign Exposure Level and Main Outcome Variables

|  | Model 1: Negative attitude towards meth use | Model 2: Ever called SASH | Model 3: Used meth in the past 30 days | Model 4: Used meth fewer than 10 days in the past 30 days | Model 5: Is considering quitting meth use |
| --- | --- | --- | --- | --- | --- |
|  | OR (95% CI) | OR (95% CI) | OR (95% CI) | OR (95% CI) | OR (95% CI) |
| Campaign exposure level |  |  |  |  |  |
| No exposure | 1 (Ref) | 1 (Ref) | 1 (Ref) | 1 (Ref) | 1 (Ref) |
| Lower-level exposure | 1.17 (0.85, 1.62) | 5.88*** (3.24, 10.66) | 2.19* (1.17, 4.09) | 1.30 (0.37, 4.53) | 0.87 (0.30, 2.55) |
| Higher-level exposure | 1.57 (1.09, 2.26)* | 7.57*** (4.16, 13.79) | 2.05* (1.07, 3.95) | 10.43*** (3.03, 35.84) | 14.16* (1.52, 131.83)^1^ |
| Last time used meth |  |  |  |  |  |
| Never | 1 (Ref) | 1 (Ref) | --- | --- | --- |
| Over 12 months ago | 1.04 (0.68, 1.59) | 5.37*** (2.51, 11.51) | --- | --- | --- |
| Over 30 days-12 months ago | 0.77 (0.43, 1.36) | 11.98*** (4.88, 29.40) | --- | --- | --- |
| In the past 30 days | 0.97 (0.59, 1.58) | 6.53*** (2.91, 14.67) | --- | --- | --- |
| Number of days used meth in the past 30 days  (Ref = 1-9 days) | --- | --- | --- | --- | 1.53 (0.48, 4.82) |
| COVID-19-related factors scale | 1.09 (0.95, 1.26) | 1.23 (0.87, 1.73) | 1.71** (1.20, 2.43) | 1.29 (0.63, 2.67) | 1.48 (0.74, 2.96) |
| MSM (Ref = No) | 1.90*** (1.35, 2.68) | 0.23*** (0.11, 0.50) | 1.66 (0.83, 3.33) | --- | --- |
| In a higher-risk job  (Ref = No) | 0.74* (0.58, 0.94) | 2.13** (1.22, 3.72) | 1.10 (0.65, 1.87) | --- | --- |
| Lives in a higher-risk zip code (Ref = No) | 0.96 (0.75, 1.24) | 1.22 (0.72, 2.08) | 1.56 (0.86, 2.83) | --- | --- |
| Unemployed at least 9 months in the past year  (Ref = No) | 1.05 (0.79, 1.40) | 1.11 (0.60, 2.03) | 3.31*** (1.99, 5.51) | --- | --- |
| Homeless in the past year  (Ref = No) | 0.98 (0.66, 1.44) | 3.57*** (1.91, 6.69) | 7.64*** (4.45, 13.13) | --- | --- |
| Age | 1.01 (0.99, 1.02) | 0.98 (0.94, 1.02) | 1.05*** (1.02, 1.08) | --- | --- |
| Gender |  |  |  |  |  |
| Female | 1 (Ref) | 1 (Ref) | 1 (Ref) | --- | --- |
| Male | 0.71* (0.54, 0.94) | 1.08 (0.60, 1.94) | 1.55 (0.76, 3.15) | --- | --- |
| Other or unsure | 1.03* (0.46, 2.29) | 2.59 (0.65, 10.28) | 4.15* (1.26, 13.69) |  |  |
| Race/ethnicity |  |  |  |  |  |
| White (non-Hispanic) | 1 (Ref) | 1 (Ref) | 1 (Ref) | --- | --- |
| Black (non-Hispanic) | 0.79 (0.55, 1.14) | 0.59 (0.25, 1.35) | 0.39* (0.15, 0.99) | --- | --- |
| Hispanic or Latinx | 0.95 (0.71, 1.27) | 0.53 (0.27, 1.03) | 0.73 (0.39, 1.35) | --- | --- |
| Asian (non-Hispanic) | 0.70 (0.47, 1.04) | 0.33 (0.10, 1.12) | 0.24 (0.05, 1.10) | --- | --- |
| Mixed race | 1.04 (0.71, 1.52) | 0.56 (0.25, 1.25) | 0.86 (0.40, 1.86) | --- | --- |
| Other (non-Hispanic) | 0.37 (0.13, 1.06) | 5.97* (1.27, 28.09) | 1.03 (0.12, 9.13) |  |  |
| Education |  |  |  |  |  |
| High school degree or less | 1 (Ref) | 1 (Ref) | 1 (Ref) | --- | --- |
| Some college or trade school | 1.33 (0.95, 1.86) | 0.54 (0.25, 1.14) | 1.29 (0.65, 2.55) | --- | --- |
| College degree | 1.59* (1.14, 2.23) | 0.52 (0.24, 1.09) | 0.73 (0.34, 1.54) | --- | --- |
| Graduate work or degree | 1.34 (0.89, 2.02) | 0.96 (0.41, 2.24) | 0.66 (0.26, 1.70) | --- | --- |
| Annual household income |  |  |  |  |  |
| ≤ $30,000 | 1 (Ref) | 1 (Ref) | 1 (Ref) | --- | --- |
| $30,001-$60,000 | 1.07 (0.80, 1.44) | 1.36 (0.66, 2.80) | 0.95 (0.51, 1.75) | --- | --- |
| $60,001 or higher | 0.85 (0.63, 1.15) | 2.75** (1.39, 5.44) | 0.55 (0.28, 1.08) | --- | --- |

*Note*. Associations were tested using multivariable logistic regression models.

OR = Odds Ratio. CI = Confidence Interval.

Model 1: Ref = Positive attitude towards meth use, *n* = 1385

Model 2: Ref = No, *n* = 1385

Model 3: Ref = No, *n* = 1385

Model 4: Ref = 10 or more days in the past 30 days, *n* = 99

Model 5: Ref = No, *n* = 96.

^1^ Large 95% CI range due to smaller sample size.

**p* ≤ .05; ***p* ≤ .01; *** *p* ≤ .001.
